# Mechanisms of natural and vaccine-induced immunity to *Bordetella pertussis*

**DOI:** 10.1371/journal.ppat.1014128

**Published:** 2026-04-24

**Authors:** Lisa Borkner, Caroline E. Sutton, Sreeram Udayan, Seyed Davoud Jazayeri, Kingston H. G. Mills

**Affiliations:** Immune Regulation Research Group, School of Biochemistry and Immunology, Trinity Biomedical Sciences Institute, Trinity College Dublin, Dublin, Ireland; University of Pittsburgh, UNITED STATES OF AMERICA

## Abstract

*Bordetella pertussis* causes whooping cough (pertussis), a respiratory infectious disease that is resurgent despite high vaccine coverage. Research on the mechanisms of immunity to *B. pertussis* have demonstrated protective roles for innate immune cells, antibodies and T cells in immunity induced by natural infection. Studies in animal models have demonstrated that IL-17-secreting respiratory tissue-resident memory CD4^+^ T (T_RM_) cells and associated recruitment of neutrophils play a critical role in clearance of bacteria from nasal mucosa. However, current acellular pertussis (aP) vaccines, while inducing potent serum antibody responses and protecting against pertussis disease, fail to induce local immune responses in the respiratory tract, thus allowing transmission of the bacteria from vaccinated individuals. Motivated by the resurgence of pertussis and the limitations of the current aP vaccines, several research groups involved in the design of more effective third generation pertussis vaccines are focusing on nasal-delivery approaches that induce respiratory T_RM_ cells and mucosal IgA, as well as circulating antibodies.

## Introduction

The Gram-negative bacterium *Bordetella pertussis* is the primary etiologic agent of whooping cough (pertussis), a severe respiratory disease, which can be fatal, especially in young infants. *B. pertussis* is transmitted by aerosol, first infecting the upper respiratory tract, before spreading to the lungs. The bacteria produce a range of toxins, including pertussis toxin (PT), which cause damage to the airways, resulting in the characteristic whooping cough disease. In most healthy individuals, the infection is controlled, and eventually eliminated, by a combination of innate and adaptive immune responses. Recovery from infection is associated with development of T and B cell memory which can prevent re-infection for years or possibly decades.

Prior to mass vaccination, pertussis was endemic, but the incidence declined significantly following introduction of the whole cell pertussis (wP) vaccines in the 1940s and had reached very low levels in most high- and middle-income countries by the 1970s. However, concerns around the reactogenicity of the wP vaccines motivated the development of safer acellular pertussis (aP) vaccines, prepared from 2-5 *B. pertussis* antigens and administered with alum as the adjuvant [1]. The aP vaccines induce high levels of circulating antibodies against the vaccine antigens. However, immunity wanes rapidly after immunization [2]. Furthermore, the aP vaccines generate Th2-polarized responses, but weak Th1 and Th17 responses, and do not generate local antibodies or T cells in the respiratory tract [3–5]. While these second-generation vaccines have moderate efficacy against severe pertussis disease, studies in animal models showed that they fail to prevent nasal infection with *B. pertussis*, thus allowing community transmission of bacteria [5–7].

The consequence of the introduction of safer, but less effective aP vaccines has been a resurgence of pertussis in many countries with high vaccine coverage, reaching epidemic levels in the last few years [2]. Part of the problem with the aP vaccines arose from a poor understanding of the mechanism of protective immunity against *B. pertussis* at the time of their design and an assumption that the generation of circulating antibody was key to protection. However, while antibodies can prevent toxin-mediated disease and are the cornerstone of effective maternal immunization programmes, they do not prevent *B. pertussis* infection, especially of the nasal mucosa. Recent studies in animal models have demonstrated that cellular immune responses, especially those mediated by tissue-resident memory T (T_RM_) cells, largely mediate protective immunity in the nasal mucosa [5]. Consequently, current efforts to develop more effective 3^rd^ generation vaccines are more strategically focused on approaches that induce T cell as well as antibody responses, especially at the site of infection in the respiratory tract.

In this article, we review the mechanisms of innate and adaptive immunity to primary infection with *B. pertussis* and how protective immune responses can be subverted by bacterial virulence factors. We discuss the relative role of T cells and antibodies in vaccine-induced protective immunity against *B. pertussis*. Finally, we describe new vaccine approaches designed to prevent infection of the nasal mucosa as well as disease, and thereby having the potential to limit community transmission of *B. pertussis*.

## Early and innate immune responses to *B. pertussis* infection

Exposure to aerosol droplets containing *B. pertussis* via the respiratory tract results in the adherence of the bacteria primarily to the ciliated cells of the trachea, bronchi, and bronchioles, resulting in descending colonization of the respiratory tract [8]. However, recent studies, using primary human nasal epithelial cells grown at the air-liquid interface, have suggested that the bacterium may initially reside and replicate within the overlying mucus layer, and only scarcely colonized the cell cilia [9]. This mucus layer may act as a protective niche, allowing the bacteria to evade early epithelial recognition and damage. Epithelial attachment is mediated by the bacterial adhesins filamentous hemagglutinin (FHA), fimbriae (Fim2 and Fim3), as well as pertactin (PRN). Colonization triggers an increase in mucus production, a decrease in epithelial barrier integrity, and disruption of mucociliary clearance by the virulence factor tracheal cytotoxin (TCT), which impairs removal of the bacteria [10,11]. Studies on human trachea bronchial or pulmonary epithelial cells have demonstrated that adenylate cyclase toxin (ACT) disrupts the tight junction integrity of the epithelial barrier and induces pro-inflammatory cytokine production, via cAMP activation [12,13].

**Fig 1:**
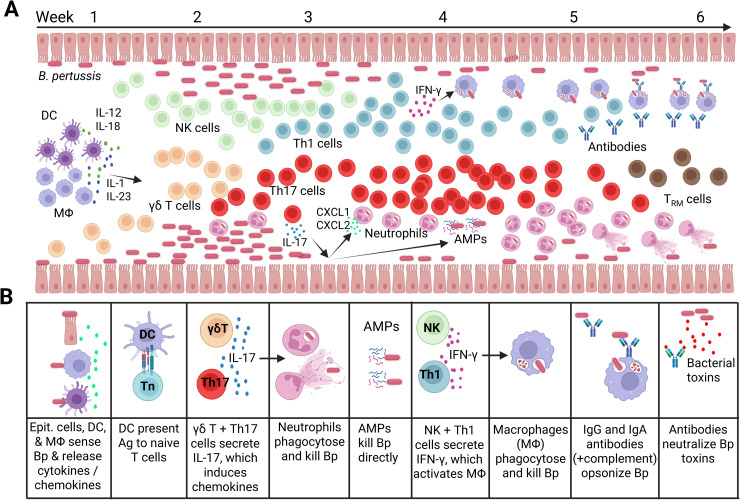
Proposed model of protective immunity to primary infection with *Bordetella pertussis* in the respiratory tract. **A)** Infiltrating and resident immune cells and molecules that control infection (based on studies in mouse models). Inhaled *B. pertussis* (Bp) binds to respiratory epithelial cells. Resident dendritic cells (DC) and alveolar macrophages (MΦ) sense the bacteria, secrete T cell polarizing cytokines and present antigens to naïve T cells (in the local lymph nodes), which differentiate into Th1 and Th17 cells (also into Treg cells that regulate immune responses and inflammation). γδ T cells and NK cells produce early IL-17 and IFN-γ, whereas Th1 and Th17 produce sustained IFN-γ and IL-17, respectively. IL-17 promotes production of AMPs, which have direct bactericidal activity, and CXCL1 and CXCL2 by epithelial cells, which recruit and activate neutrophils that phagocytose and kill *B. pertussis*. IFN-γ activates macrophages, which phagocytose and kill *B. pertussis.* Uptake of *B. pertussis* by macrophages is enhanced by *B. pertussis-*specific antibodies (IgG and IgA). Antibodies also neutralize *B. pertussis* toxins. Infiltrating Th1 and Th17 cells become resident T_RM_ cells that persist in the respiratory tract poised to respond rapidly to re-infection. **B)** Immune effector cells and modules that mediate protective immunity against *B. pertussis* infection of the respiratory tract. *Created in BioRender. Mills, K. (2026)* https://BioRender.com/x9wlb9l.

The early host response to *B. pertussis* involves recruitment and activation of cells of the innate immune system, which act in synergy with the epithelium to induce chemokines required for further cellular recruitment [14]. Innate cell-derived IFN-γ and IL-1β, as well as IL-17 production from T cells, help to drive chemokine production from human epithelial cells in response to *B. pertussis* [10,15]. In mice *B. pertussis* infection triggers sequential recruitment of dendritic cells (DCs), macrophages and γδ T cells to the respiratory tract, followed by neutrophils, natural killer (NK) cells, and finally, αβ T cells, predominantly CD4^+^ T cells [16,17] (Fig 1).

### Macrophages

Animal models of *B. pertussis* infection and numerous *in vitro* studies have revealed that *B. pertussis* can enter, survive, and persist in macrophages. *B. pertussis* has been found in pulmonary alveolar macrophages from infants [8]. The persistence and replication of *B. pertussis* in macrophages is facilitated by PT and ACT, which downregulate the inflammatory and bactericidal responses of the macrophages. ACT can suppress chemokine and pro-inflammatory cytokines, while increasing the production of IL-10 from human macrophages [18]. ACT can also induce apoptosis of alveolar macrophages, whereas PT can interfere with recruitment and inflammatory signaling in macrophages [19,20].

Significant numbers of Ly6C^+^ macrophages are recruited to the nose and lung of mice by day 7 of infection with *B. pertussis* [15]. Bacterial recognition, either through phagocytosis or recognition of bacterially derived TLR agonists, drives the induction of pro-inflammatory responses by macrophages in the respiratory tract [21]. However, there is increased expression of anti-inflammatory pathways in macrophages infected with *B. pertussis*, suggesting that this may be a bacterial survival strategy. Therefore, subversion of the macrophage-driven anti-microbial responses is essential for survival of the bacteria in the host.

### Dendritic cells

Resident and infiltrating immature DCs in the respiratory tract sense pathogen-associated molecular patterns (PAMPs) and virulence factors from *B. pertussis*. Activation of TLR4 by *B. pertussis* lipopolysaccharide (LPS) promotes DC maturation and the secretion of IL-1β, IL-12, and IL-23, which drives the induction of Th1 and Th17 cells [22,23]. However, FHA and *B. pertussis* LPS induce the anti-inflammatory cytokine IL-10 from DCs, which can promote the induction of regulatory T (Treg) cells that suppress protective Th1 cell responses against *B. pertussis* [24,25].

*B. pertussis* has developed a range of strategies to subvert DC function. PT inhibits migration of infected DCs from the lungs into the draining lymph nodes, thus suppressing activation of T cells and facilitating bacterial persistence [26]. ACT inhibits IL-12p35 production by human DCs by enhancing levels of intracellular cAMP concentrations and thereby suppresses development of Th1 cells [27]. However, ACT also promotes the activation of caspase-1 and the NLRP3 inflammasome, required for processing pro-IL-1β to its mature form [28]. The resulting IL-1β, together with IL-23, promotes expansion of Th17 cells. Thus, it appears that *B. pertussis* virulence factors can both stimulate as well as inhibit DCs that drive protective Th1 and Th17 responses.

### Neutrophils

Neutrophils represent the largest population of immune cells recruited to the lungs and nasal tissues in the first week of infection and peak 10–14 days after aerosol challenge of mice with *B. pertussis* [15]. Initial binding of *B. pertussis* to the surface of neutrophils is facilitated by FHA [29], and this binding further enhances the expression of the complement receptor 3, the receptor for the bacterial toxin ACT [30]. Neutrophil recruitment is activated by chemokines induced by IL-17-producing Th17 and γδ T cells [15]. A novel subset of neutrophils that express Siglec-F accumulate in the nasal tissue during *B. pertussis* infection of mice [15]. Depletion of neutrophils or blocking IL-17 *in vivo* significantly delayed clearance of *B. pertussis* from the nasal mucosa. IL-17, together with IFN-γ can also enhance neutrophil-induced killing of *B. pertussis* [31]. Complement-deficient (C3^−/−^) mice fail to recruit neutrophils and have reduced bacterial clearance [32]. Opsonizing antibodies from convalescent pertussis patients were shown to increase reactive oxygen species (ROS) production and killing of *B. pertussis* by neutrophils [33].

Neutrophil recruitment and function during *B. pertussis* infection is limited through FHA, PT, and ACT [34–36]. ACT potently inhibits neutrophil activation, limiting its ability to induce ROS, via increased intracellular cAMP levels, as well as reducing the expression of complement and FcγRs [34]. PT suppresses CXCL1 and CXCL2-induced recruitment of neutrophils in neonatal mice [32]. PT also induces type III interferon by neutrophils, which limits IFN-γ production, leading to increased bacterial loads and inflammation in the lungs [37].

### NK cells

NK cells are recruited to the lungs early in infection of adult mice with *B. pertussis* and provide a source of IFN-γ before the induction of Th1 cells [17]. The early burst of IFN-γ produced by NK cells is essential for limiting the bacterial load in the lungs [17]. Infant mice have immature NK cells that produce less IFN-γ and have fewer pulmonary-infiltrating NK cells, leading to disseminating and often lethal infection with *B. pertussis* [38].

Human NK cells do not respond directly to *B. pertussis*; however, they produce IFN-γ in the presence of macrophage-derived IL-1β and IL-18 [39]. Recent studies on human airway epithelial cells (HAE) have shown that NK cells cooperate with epithelial cells and innate immune cells to limit infection [40]. NK cell-derived IFN-γ along with innate immune cell activation is crucial for secretion of CXCL9 and CXCL10 from HAE, resulting in further immune cell recruitment to the site of infection [39,40].

### Antimicrobial peptides (AMPs) and complement

AMPs produced by epithelial cells, neutrophils, and macrophages, in response to IL-17 and IL-22, have potent microbicidal activities. The AMPs granulysin, cathepsins, and defensins are produced in response to *B. pertussis* infection; α-defensin-1 and -5 can limit the toxicity of PT [41].

Complement plays a role in *B. pertussis* clearance through opsonization and direct killing of bacteria [42]. Once activated, the complement pathway drives membrane attack complex (MAC) formation and targeted destruction of the pathogen. Studies with human sera from previously infected individuals have shown that antibodies against LPS and pertactin are bactericidal, particularly LPS-specific IgG3 [43].

*B. pertussis* employs a sophisticated array of surface proteins to resist complement-dependent killing [44]. This includes subversion of the classical complement pathway through production of Bordetella resistance to killing protein (BrkA), which confers resistance by physically interfering with the deposition of C4, C3, and C9 components, thereby limiting the formation of the MAC, and limiting subsequent neutrophil-mediated phagocytosis [45]. FHA, virulence-associated gene 8 (Vag8), and Bordetella polysaccharide (Bps) can also aid the survival of the bacterium by limiting the deposition of complement antibody onto the surface of the bacterium [46–48]. Bordetella also targets the alternative pathway through the binding of Factor H, which sequesters it away from binding to C3B [49]. The evasion of the complement pathway is essential for the survival of *B. pertussis* and host colonization.

## Adaptive immunity induced by infection

### T cells

Mechanistic experiments in animal models, backed up with more observational studies in humans, have provided definitive evidence of a role for CD4^+^ T cells in protective immunity against primary and subsequent infections with *B. pertussis*. The first study to identify a protective role for cellular immunity established that IFN-γ-producing CD4^+^ T cells (Th1 cells) are induced during infection of mice with *B. pertussis* and that adoptive transfer of Th1 cells from *B. pertussis* convalescent mice to naïve irradiated immunosuppressed recipients conferred protection against *B. pertussis* challenge [50]. Furthermore, mice lacking the IFN-γ receptor failed to clear the bacteria, which disseminated from the respiratory tract [51].

Studies in baboons and humans have identified long-lived *B. pertussis*-specific CD4^+^ T cells [52–54]. Peripheral blood CD4^+^ T cells in infants and children recovering from whooping cough produced IFN-γ in response to stimulation with *B. pertussis* antigens [53]. Circulating CD4^+^ T cells that produce IL-17 are also induced during *B. pertussis* infection of mice [31] and baboons [52].

A key limitation of many studies on T cell responses to *B. pertussis* is that they focused on peripheral T cells, rather than on T cells at the site of infection in the lung or nasal mucosa. However, studies in mice, which enabled discrimination of tissue-resident from circulating immune cells, have shown that CD4^+^ T cells that express CD44, CD69, with or without CD103, and secrete IL-17 or IFN-γ in response to *B. pertussis*, accumulate in the lung and nasal tissue as T_RM_ cells during infection and persist after bacterial clearance [15,55]. *B. pertussis*-specific CD4^+^ T_RM_ cells are maintained in the tissue by bystander re-activation with unrelated pathogens and are first responders to re-infection with *B. pertussis* [56]. Re-activation of T_RM_ cells mediates rapid clearance of *B. pertussis* from the lungs and nasal mucosa following re-challenge of convalescent mice [15,55]. *B. pertussis* infection also promotes recruitment of IL-17-secreting γδ T cells with a tissue-resident memory phenotype and these cells contribute to the pool of IL-17 that mediates bacterial clearance [57]. Studies involving IL-17^−/−^ mice or treatment of mice with depleting or blocking antibodies specific for CD4 or IL-17 have provided definitive evidence of a non-redundant role for *B. pertussis*-specific IL-17-secreting CD4⁺ T_RM_ cells in clearance of primary and secondary infections of the nose with *B. pertussis* [15]. The protective role of IL-17 is mediated through induction of the chemokines CXCL1 and CXCL2, which promote recruitment of Siglec-F^+^ neutrophils, which together with AMPs, promote bacterial clearance from the nasal mucosa [15].

In addition to effector T cells, *B. pertussis* infection also induces IL-10-secreting Treg cells, which suppress IL-17 and IFN-γ production by CD4^+^ T cells [24,58]. This may serve as a strategy evolved by the bacteria to subvert protective cellular immune responses and thereby prolong infection of the host.

### B cells and antibodies

It is well established that systemic IgG and mucosal IgA responses are induced during *B. pertussis* infection, however, few studies have explored the precise contribution of B cells and antibodies in conferring protective immunity to infection. Transcriptomic profiling of lungs of mice after *B. pertussis* infection revealed upregulation of several B cell associated genes, including the B cell chemoattractant, CXCL13, and immunoglobulin genes involved in the formation of IgG, IgA, and IgM [14]. Mice lacking B cells fail to clear *B. pertussis* from nasal cavity, trachea, and lungs. Although there may be a role for B cells outside of antibody protection, the results from adoptive transfer studies involving colostrum from convalescent mice or humans demonstrated a protective role for antibodies [59]. Antibodies facilitate *B. pertussis* killing by opsonization and subsequent phagocytosis through Fc receptors, FcαR and FCγR [35,60]. In addition, *in vitro* studies have suggested that antibodies directed against *B. pertussis* fimbriae can prevent bacterial adherence to ciliated respiratory epithelial cells [61]; however, it is not known if this mechanism operates *in vivo*.

## Immunity induced with licensed pertussis vaccines

### Whole-cell pertussis vaccines

wP vaccines consist of chemically inactivated *B. pertussis* bacteria, presenting a repertoire of antigens and PAMPs that activate a broad range of immune pathways. The wP vaccine triggers strong TLR4-dependent activation of monocytes and DCs, leading to the production of inflammatory and T cell polarizing cytokines, which direct the induction of Th1 and Th17 cells, and the development of CD4^+^ T cell memory [23]. Immunization of mice with wP vaccines induces antigen-specific IL-17- and/or IFN-γ-secreting CD4^+^ T_RM_ in the lung and nasal cavity, which subsequently expand locally following infection with *B. pertussis* and mediate rapid clearance of bacteria from lungs and nasal mucosa by activating neutrophils and macrophages [5,15].

Although studies on respiratory T_RM_ cells are more difficult in humans, a recent investigation that utilized immune cells from tonsil or by nasal swabbing of adult volunteers, revealed that adults primed with wP vaccines during childhood exhibited significantly higher frequencies of IL-17A and IFN-γ-producing respiratory T_RM_ cells [4]. These antigen-specific T_RM_ cells persisted for decades post-vaccination, suggesting that wP vaccines induce long-lived T cell responses in the respiratory tract [4].

Despite greater immunogenicity, especially for T cell responses, and high protective efficacy when compared with aP vaccines, traditional wP vaccines are associated with higher reactogenicity, including fever and occasional seizures [62]. A reduced reactogenicity wP (RRwP) vaccine was recently developed where the *lgmB* gene is deleted, resulting in a modified Lipid A that reduces TLR4 activation by *B. pertussis* lipooligosaccharide, the gene encoding dermonecrotic toxin (DNT) is deleted, and PT is genetically inactivated [63]. Intramuscular (i.m.) immunization of baboons with the RRwP vaccine induced robust Th1/Th17-skewed cellular responses, comparable to those elicited by conventional wP vaccines, and generated high IgG titers [62]. The RRwP vaccine was less reactogenic but retained the ability to prevent disease and reduce bacterial colonization of the nasopharynx after challenge with *B. pertussis* [62].

### Acellular pertussis vaccines

aP vaccines, consisting of detoxified PT and up to four additional antigens, FHA, PRN, FIM2, and FIM3, usually in combination with diphtheria and tetanus toxoid and alum as the adjuvant were developed in the 1980s in response to concerns regarding the reactogenicity of wP vaccines. The aP vaccines were safer and had an efficacy of 84% or 85% against severe pertussis disease, compared with up to 95% for wP vaccines [1]. Protection induced with aP vaccines was associated with strong PT neutralizing antibodies [64].

Antibodies specific for PRN have phagocytic [65] and bactericidal [66] activity and confer protection against lung infection in a mouse model [67]. The emergence of PRN-deficient strains due to aP vaccine-induced immune pressure has been documented in countries relying on aP vaccines containing PRN [68].

Parenterally administered aP vaccines generate high serum antibody titers but fail to induce mucosal sIgA [69], which prevents *B. pertussis* from adhering to endothelial cells and facilitates opsonophagocytosis of the bacteria early in the infection. Furthermore, studies in mice and baboons have shown that aP vaccines induce Th2 responses, but have limited ability to induce Th1 and Th17-type respiratory T_RM_ cells [3,5,7] Furthermore, aP vaccines can suppress the induction of IL-17-secreting T_RM_ cells and enhance nasal carriage of *B. pertussis* [6], in part by inducing IL-10-secreting regulatory CD4 and CD8 T cells [70].

Consistent with the studies in animal models, a recent study in humans showed that adult volunteers who had been immunized with aP as children had weak IL-13-secreting, but undetectable IL-17 or IFN-γ-secreting, *B. pertussis*-specific respiratory T_RM_ cells [4]. The limited ability of aP vaccines to induce systemic Th1 and Th17 cells, respiratory T_RM_ cells, or mucosal IgA may explain their failure to prevent community transmission of *B. pertussis*, leading to a resurgence of pertussis in many countries with high vaccine coverage [2]. Furthermore, the emergence of *B. pertussis* strains with attenuated expression or deletion of antigens contained in the aP vaccine (in particular PRN), through vaccine-driven immune selective pressure, may have facilitated the persistence of *B. pertussis* in populations immunized with aP vaccines [71,72].

## Immunity induced with experimental pertussis vaccines

### Protein subunit vaccines with novel adjuvants

The first and simplest approach assessed to improve aP vaccine efficacy was to add a more potent adjuvant to the existing alum-adjuvanted aP vaccine or to an experimental vaccine that includes the existing aP vaccine antigens. Many of the early studies on novel adjuvants were based on TLR agonists, including the TLR4 agonists MPLA [73], LPxL1 [74], LPxL2 [73] and BECC438b [75], the TLR9 agonist, CpG [31], the TLR2 agonist LP1569 [76], and the TLR7/8 agonist SMIP7.10 [77] (Table 1). These experimental vaccines were delivered parenterally and all induced systemic antibody responses and some promoted Th1 responses and had improved protective efficacy against lung infection in mice. However, these studies did not assess protection against nasal infection and did not examine the induction of respiratory T_RM_ cells.

**Table 1:** Vaccine induced immunity to *Bordetella pertussis.*

Vaccine	Species	Route	Serum IgG	Mucosal IgA	Systemic T cells	Respiratory T_RM_ cells	Protection lungs	Protection nose	Ref(s)
wP vaccine	Mouse	IP	+++^1^	–	Th1/Th17	++	+++	++	[3,5]
	Baboon	IM	+++	–	Th1/Th17	NT	NT	++	[7,105]
	Human	IM	+++	NT	Th1/Th17	+	NT	NT	[4]
aP vaccine	Mouse	IP	++++	–	Th2	–	++	–	[3,5]
	Baboon	IM	++++	–	Th2	NT	NT	–	[7]
	Human	IM	++++	NT	Th2	–	NT	NT	[4]
	Rat	IM	+++	–	NT	NT	+	+	[78]
		IN	+++	+	NT	NT	+	+	
		OG	–	–	NT	NT	+	+	
**Attenuated vaccines**									
BPZE1	Mouse	IN	+++	+++	Th1/Th17	+++	+++	+++	[69]
	Baboon	IN	+++	NT	NT	NT	NT	++	[91]
	Human	IN	+++	++	NT	NT	NT	NT	[93,94]
Bbvac	Mouse	IN	+++	NT	Th17	+	+++	+++	[95]
**Particulate vaccines**									
OMV	Mouse	SC	+++	–	Th1	–	+++	+	[86,87]
		Aer./IN	+++	+++	Th1/Th17	+	+++	++	
OMV	Mouse	IP	NT	NT	Th1/Th17	+++	+++	NT	[88]
OMV	Mouse	IM	+++	–	Th1/Th17	NT	+++	+	[89]
		IN	+	+++	Th1/Th17	NT	+++	++	
AIBP	Mouse	Aer./IN	+++	+++	Th1/Th17	+++	+++	+++	[96]
**Protein vaccines + novel adjuvants**									
aP + MPLA	Mouse	SC	++++	NT	↓Th2	NT	+++	NT	[73]
aP + LPxL2	Mouse	SC	++++	NT	↓Th2	NT	+++	NT	[73]
aP + alum + BECC4386	Mouse	IN	++++	++	NT	NT	+++	+	[75]
		IM	++++	–			+++	+	
aP + alum + BcfA	Mouse	IM	+++	–	–	+	+++	++	[80]
		IM/IN	++	+++	Th1/Th17	+++	+++	++	
aP + alum + CpG	Mouse	IP	++++	NT	Th1/Th17	NT	+++	NT	[106–108]
		IN	++++	+	NT	NT	+++	NT	
aP + alum + CpG 1018	Mouse	IM	++++	NT	NT	NT	+++	+	[109]
	Human	IM	++++	NT	NT	NT	NT	NT	[110]
aP + CpG	Mouse	IP	++++	NT	Th1/Th17	NT	++	NT	[31,106]
aP + curdlan	Mouse	IN	++++	++	Th2/Th17	–	+++	++	[79]
		IP	++++	–	Th2/Th17	–	+	++	
aP + alum + SMIP7.10	Mouse	IM	++++	NT	Th1/Th17	NT	+++	NT	[77]
aP + LP1569	Mouse	IP	+++	NT	Th1/Th17	NT	+++	NT	[76]
aP + c-di-GMP	Mouse	IN	+++	+++	Th1/Th2/ Th17	+++	+++	+++	[82]
aP + LP-GMP	Mouse	IP	+++	+	Th1	+	+++	+	[81]
		IN	+++	+++	Th17	+++	+++	++	
aP + alum + LP-GMP	Mouse	IN	++	++	Th1/Th17	+++	NT	++	[70]
		IM	+++	–	Th1	–	NT	–	
aP + T-vant	Mouse	IN	+++	+++	NT	+++	+++	+++	[83]
**mRNA vaccines**									
DTP mRNA (10 Ag)	Mouse	IM	+++	NT	Th1	NT	++	+	[84]
	Rat	IM	+++	NT	NT	NT	++	+	[85]

^1^Magnitude of immune responses shown in an arbitrary scale as: −, + , ++, +++ and ++++; IP, intraperitoneal; IM, intramuscular; IN, intranasal, SC, subcutaneous; OG, oral gavage; NT, not tested, Ag, antigen; Aer, aerosol. Ref(s), reference/references.

Following the discovery that parenterally delivered aP vaccines do not prevent nasal infection with *B. pertussis*, the emphasis on new pertussis vaccine design shifted to intranasal (i.n.) delivery of experimental vaccines with mucosal adjuvants to induce respiratory T_RM_ cells and mucosal IgA. In a coughing rat model of pertussis, immunization with DTaP by i.m., i.n., or oral routes decreased the *B. pertussis* bacterial burden in the respiratory tract and protected against *B. pertussis*-induced cough [78]. Immunization by the i.m and i.n., but not oral, routes also protected against respiratory distress. Addition of BECC438b, a TLR4 agonist from *Yersinia pestis*, to DTaP (Infanrix) and delivery to mice by the nasal route enhanced bacterial clearance from lung and trachea after *B. pertussis* challenge, but did not enhance protection against nasal infection when compared with DTaP alone [75]. Furthermore, i.n. delivery of DTaP, with or without the dectin-1 agonist curdlan, induced IgA and enhanced IL-17 production in the lung and conferred greater protection against lung infection than the equivalent vaccines administered by the i.p. route [79]. However, protection against nasal infection was similarly modest with the DTaP vaccine administered by the i.n. or i.p. routes with or without curdlan. An i.m. prime/i.n. pull immunization approach with Tdap (Boostrix) supplemented with BcfA, an outer membrane protein and TLR4/TLR2 agonist from *Bordetella bronchiseptica,* promoted respiratory IL-17-secreting T_RM_ cells and protected against infection of the lung and significantly improved protection in the nose compared to Tdap alone [80].

Agonists for stimulator of interferon genes (STING), which sense bacterial and host-derived cyclic dinucleotides and promotes innate immune responses, have also been shown to be effective adjuvants for pertussis subunit vaccines. A combination of the STING agonist c-di-GMP and LP1569, a TLR2 agonist from *B. pertussis*, called LP-GMP, was an effective adjuvant for parenteral and nasal delivery of an experimental aP vaccine, composed of genetically detoxified PT, FHA, and PRN or when added to a commercial Tdap vaccine (Boostrix) [70,81]. Delivery of the experimental vaccine with LP-GMP as the adjuvant by the i.n. route induced potent IL-17 and IFN-γ-secreting T_RM_ cells in the lung and nasal tissues and conferred good protection against *B. pertussis* infection of the lung and nose [81]. In a subsequent study, i.n. delivery of a 3-component experimental aP vaccine formulated with STING agonists alone induced Th1/Th2/Th17 response in the spleen cells and IgA in nasal wash and protected against *B. pertussis* infection of the lung, trachea, and nose [82]. Surprisingly, this experimental aP vaccine administered i.n. without the adjuvant also conferred significant protection against upper and lower respiratory tract infection [82].

Finally, three i.n. immunizations with an experimental aP vaccine adjuvanted with outer membrane vesicles (OMV) from *Burkholderia pseudomallei,* called T-vant, induced IgA, IgG, Th1, Th2, and Th17 CD4^+^ T cells at the mucosal sites and protected *B. pertussis* infection of the lungs and nasopharynx [83].

### mRNA pertussis vaccines

mRNA vaccines, comprising synthetic mRNA, which directs the synthesis of one or more antigens, often encapsulated by lipid nanoparticles, have the potential to be a fast, adaptable, and potent tool against infectious diseases. Preclinical studies with DTP mRNA vaccine formulations containing up to 10 antigens from *B. pertussis* administered i.m. to mice [84] or rats [85] induced antibody responses against each of the antigens [84,85], and when compared with an alum-adjuvanted aP vaccine, generated stronger Th1 responses in the spleen [84]. The pertussis mRNA vaccines protected against lung infection following challenge with classical and contemporary strains of *B. pertussis* [84,85] and protected against *B. pertussis*-induced cough in rats [85]. While mRNA-based pertussis vaccines have promise, they may be more efficacious in preventing nasal infection if optimized for delivery by the nasal route.

### Pertussis outer membrane vesicle (OMVs) vaccines

OMVs are nano-sized vesicles that naturally shed from the bacterial outer membrane that carry a diverse array of native antigens and PAMPs, enabling them to stimulate both innate and adaptive immune responses. Pulmonary or i.n. immunization of mice with *B. pertussis* OMV vaccines elicited robust mucosal IgA production and Th1/Th17 responses in spleen and pulmonary tissues and conferred protection against infection of the lungs and trachea, and reduced the bacterial load in the nasal cavity following challenge with *B. pertussis* [86,87]. When compared with subcutaneous immunization, i.n. delivery of the OMV vaccine induced mucosal IgA and stronger antigen-specific respiratory CD4⁺ T_RM_ cells and provided superior protection against infection of the upper respiratory tract [87]. Furthermore, *B. pertussis* OMV vaccines significantly outperformed aP vaccines, inducing Th1 and Th17 responses and protecting against *B. pertussis* infection of the lungs [88] and reducing bacterial colonization in the upper respiratory tract [89].

### Attenuated pertussis vaccines

The attenuated *B. pertussis* vaccine, BPZE1, where DNT has been deleted, TCT expression reduced, and PT genetically detoxified [90], has undergone extensive testing in animal models and phase 1 and 2 clinical trials in humans. Intranasal immunization of mice with BPZE1 induced secretory IgA as well as Th1/Th17-type T_RM_ cells in the nasal mucosa, and protected mice against infection of the lung and nose with a virulent strain of *B. pertussis* [69]. In baboons, BPZE1 transiently colonized the nasopharynx and induced FHA-, PT-, and PRN-specific IgG and IgA, protected against disease, and significantly reduced bacterial load in the nasopharynx following challenge with a virulent strain of *B. pertussis* [91]. In a phase 1 clinical trial, immunization of humans with BPZE1 enhanced *B. pertussis*-specific serum IgG and IgA responses in colonized subjects. Non-colonized subjects had significantly higher pre-challenge antibody titers against FHA, pertactin, and fimbriae, suggesting that existing *B. pertussis*-specific immune responses can prevent replication of BPZE1 [92]. A phase 2a trial showed that administration of BPZE1, using a mucosal atomization device, led to antigen-specific serum IgG and IgA production in 73% of recipients at the highest dose [93]. BPZE1 induced mucosal IgA and protected against re-challenge with BPZE1, whereas immunization with Tdap did not protect against infection with BPZE1 [94].

An alternative attenuated pertussis vaccine, Bbvac, prepared from a *B. bronchiseptica* strain lacking the sigma factor *btrS,* which increases expression of virulence factors, has been tested in pre-clinical studies. Immunization of mice with Bbvac induced long-lasting anti-bordetellae serum IgG, Th17 responses in the lung, and protected against infection of the lung and nose with *B. bronchiseptica*, *B. pertussis*, or *B. parapertussis* [95].

### Antibiotic-inactivated *B. pertussis* (AIBP) vaccine

We have recently developed a new vaccine approach based on respiratory delivery of antibiotic-inactivated bacteria. *B. pertussis* treated with fluoroquinolone antibiotics (ciprofloxacin or levofloxacin), where the bacteria are inactivated, and sometimes enlarged but not lysed, potently activates antigen-presenting cells to drive Th1 and Th17 responses [96]. Immunization of mice with the AIBP vaccine by aerosol or i.n. administration induced sterilizing immunity against lung and nasal infection. The level of protection vastly exceeded that induced with an aP vaccine and was also substantially better than that generated with the wP vaccines delivered by i.m. or i.n. routes. Importantly, protection against lung or nasal infection with *B. pertussis* induced by the AIBP vaccine was not blunted by prior parenteral immunization with an aP vaccine that induces Th2 and Treg cells [96]. A single dose of the AIBP vaccine induced IL-17 and IFN-γ-secreting T_RM_ cells in the nose and lungs, whereas two doses were required to generate *B. pertussis*-specific mucosal IgA and serum IgG1 and IgG2c.

Mechanistic studies demonstrated that protection against nasal infection was largely mediated by IL-17-secreting CD4^+^ T cells and associated recruitment of Siglec-F^+^ neutrophils to the nasal tissues [96]. Respiratory immunization with the AIBP vaccine did not promote systemic pro-inflammatory responses, unlike a parenterally delivered wP vaccine, which induced high levels of serum IL-1β, IL-6, TNF, and C-reactive protein. These findings suggest that the AIBP vaccine is a safe and effective respiratory-delivered vaccine platform for inducing T cell-mediated protective immunity against *B. pertussis* infection of the upper and lower respiratory tract.

### Maternal vaccination

Infants receive their first dose of DTaP at 2 or 3 months, leaving them vulnerable to infection during the first months of life. However, maternal antibodies induced by vaccination during pregnancy can be transferred to the child via the placenta and breast milk, providing passive immunity against disease. A systematic review of randomized controlled trials and real-world observations showed that the incidence of pertussis was significantly lower in infants born to mothers that received a booster dose of an aP vaccine during pregnancy [97]. Studies in baboons demonstrated that the offspring of baboons born to mothers vaccinated with a monocomponent PT vaccine during pregnancy were protected against clinical disease despite bacterial colonization of the lung [98], demonstrating that anti-PT antibodies are central to protection against pertussis disease.

A concern around maternal vaccination is immune blunting, where maternally derived antibodies reduce the infant’s immune responses to their primary series of pertussis vaccinations. Infants of immunized mothers typically show significantly lower antibody titers, particularly against PT, and other antigens in the DTaP vaccine [97,99], and generally “catch up” immunologically to those born to unvaccinated mothers when they receive the booster dose at 12 or 15 months of age [97,100]. However, it is not clear that this has a major impact on protection against pertussis, tetanus, or diphtheria, with two studies showing absence of clinically significant blunting in older, vaccinated infants [101,102]. Maternal vaccination programs have been implemented for a little more than a decade and the effects of immune blunting need to be studied further, but at this time, it appears that the trade-off of potentially reduced protection between 4 and 6 months is offset against enhanced protection against potentially fatal pertussis in the first 3–4 months of life. Overall, there is a significant benefit of maternal immunization in reducing the incidence of pertussis in infants [97].

## Conclusions and future perspectives

Since the introduction of aP vaccines in the 1990s, considerable progress has been made in our understanding of the mechanisms of immunity to *B. pertussis*. Quantification of serum antibodies were almost the sole focus of studies to determine vaccine immunogenicity before and during the phase 3 clinical trials of the current aP vaccines [1]. However, these and follow-up studies failed to find a serological correlate of protective immunity. Following the resurgence of pertussis in countries with high aP vaccine coverage, together with data emerging from animal models showing that T cells played a key role in natural and vaccine immunity to *B. pertussis* [50], the pertussis research community started to take cellular immunity more seriously. Initially the focus was on the protective role of Th1 cells in clearance of *B. pertussis* from the lungs through activation of macrophages [51]. Early attempts to design safe but more effective pertussis vaccines were still focused on antibody responses, but many now include evaluation of systemic T cell responses (Table 1).

Two significant advances were made in recent years that represent a paradigm shift in our understanding of immunity to *B. pertussis*. Firstly, studies in the baboon model showed that immune responses induced with current aP vaccines, while preventing pertussis disease, did not prevent nasal infection and allowed transmission of the bacteria [7]. Secondly, mechanistic studies in the mouse model showed that local IL-17-secreting T_RM_ cells mediated clearance of *B. pertussis* from the nasal mucosa [15]. Furthermore, a failure of aP vaccines to prevent nasal infection in mice was associated with the failure of this parenterally-delivered vaccine to induce respiratory T_RM_ cells [5,6]. These findings in mice, validated by studies in the baboon [52] and humans [4], not only established IL-17-secreting T_RM_ cells as a correlate of protective immunity against *B. pertussis* in the nasal mucosa, but also changed the focus of most future vaccine studies.

The induction of T_RM_ cells in lungs and nasal tissue is greatly facilitated by nasal delivery of the vaccine. Indeed, the best protection against nasal infection with *B. pertussis* is conferred by immunization approaches that induce IL-17-secreting respiratory CD4^+^ T_RM_ cells and these can be induced by i.n. vaccines based on attenuated bacteria [69], antibiotic-inactivated bacteria [96], OMVs [83,87] or soluble antigens with potent mucosal adjuvants [81]. While IFN-γ plays a key role in activating macrophages that help to clear bacteria from the lungs, antibodies, especially mucosal IgA, facilitate opsonization and prevent bacterial adherence to respiratory cells, IL-17 is critical for recruitment of neutrophils and induction of AMPs, which protect the upper respiratory tract [15,69,83].

**Fig 2:**
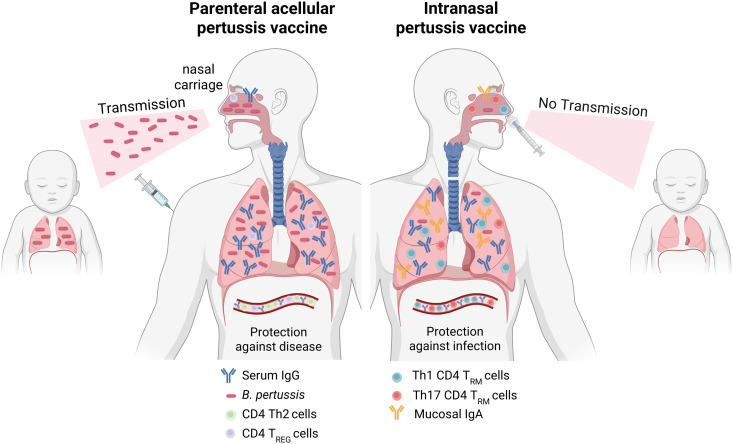
Immunity induced with parenterally-delivered aP vaccines versus next-generation mucosal pertussis vaccines. Parenteral immunization with current aP vaccines induces circulating IgG,Th2 and Treg cells, and prevents pertussis disease (through antibody-mediated neutralization of *Bordetella pertussis* toxins). However, aP vaccines do not induce mucosal antibodies or Th1/Th17-type T_RM_ cells in the respiratory mucosa and allow transmission of *B. pertussis* to naive or even immunized hosts. In contrast, intranasally delivered pertussis vaccines based on attenuated bacteria, antibiotic-inactivated bacteria, OMV, or soluble antigens with potent mucosal adjuvants, generate IgA and Th1/Th17-type T_RM_ cells in the lung and nasal tissue, as well as serum IgG. These responses protect against infection and prevent transmission of *B. pertussis*. *Created in BioRender. Mills, K. (2026)* https://BioRender.com/ng9p113*.*

Next-generation vaccines against pertussis are likely to be respiratory-delivered immunogens that include multiple *B. pertussis* antigens and endogenous PAMPs, which potently promote induction of IL-17-secreting respiratory CD4^+^ T_RM_ cells, but are also safe for use in humans, including infants. Cases of Bell’s palsy have been documented following i.n. immunization with an influenza vaccine containing *E. coli* heat-labile enterotoxin (LT) as the adjuvant [103]. However, studies in mice have shown that i.n. delivery of the closely related cholera toxin (CT) can induce proinflammatory responses in the olfactory bulbs and brain [104]. LT and CT bind to gangliosides, which are highly expressed on neuronal cells. This suggests that the toxicity associated with intranasally-delivered influenza vaccines was mediated by the adjuvant used rather than the route of delivery. There are indications that higher doses of antigens may be required for i.n. delivered protein vaccines with adjuvants, which would increase production costs, this is less likely to be a limitation of the AIBP or attenuated pertussis vaccines. While the introduction of new pertussis vaccines, especially for pediatric use, will not be logistically straightforward, they do have the potential to prevent nasal colonization and community transmission of *B. pertussis* (Fig 2). The first step will probably be a stand-alone booster pertussis vaccine for adults and adolescents, but these need to be potent enough to overcome the Th2-polarizing and suppressive immune responses of previously administered aP vaccines.

**Key learning points**

- Innate and adaptive immune responses control infection with *B. pertussis*, but these responses can be subverted by key *B. pertussis* virulence factors.
- IL-17-secreting tissueresident memory CD4 T (T_RM_) cells mediate adaptive immunity to *B. pertussis* in the nasal mucosa.
- Current acellular pertussis vaccines fail to induce local T cell or antibody responses in the respiratory tract or to prevent nasal infection with *B. pertussis*, allowing transmission of the bacteria from vaccinated individuals.
- There is a resurgence of pertussis in many countries with high vaccine coverage.
- Induction of local immune responses, especially CD4 T_RM_ cells, in the respiratory tract is key to the design of more effective pertussis vaccines that prevent *B. pertussis* infection of the lungs and the nose.

**Top five papers**

1. Borkner L, Curham LM, Wilk MM, Moran B, Mills KHG. IL-17 mediates protective immunity against nasal infection with *Bordetella pertussis* by mobilizing neutrophils, especially Siglec-F(+) neutrophils. Mucosal Immunol. 2021;14(5):1183–202.
2. Warfel JM, Zimmerman LI, Merkel TJ. Acellular pertussis vaccines protect against disease but fail to prevent infection and transmission in a nonhuman primate model. Proc Natl Acad Sci U S A. 2014;111(2):787–92.
3. McCarthy KN, Hone S, McLoughlin RM, Mills KHG. IL-17 and IFN-γ-producing respiratory tissue-resident memory CD4 T Cells persist for decades in adults immunized as children with whole-cell pertussis vaccines. J Infect Dis. 2024;230(3):e518–e23.
4. Jazayeri SD, Borkner L, Sutton CE, Mills KHG. Respiratory immunization using antibiotic-inactivated *Bordetella pertussis* confers T cell-mediated protection against nasal infection in mice. Nat Microbiol. 2025;10(12):3094–106.
5. Keech C, Miller VE, Rizzardi B, Hoyle C, Pryor MJ, Ferrand J, et al. Immunogenicity and safety of BPZE1, an intranasal live attenuated pertussis vaccine, versus tetanus–diphtheria–acellular pertussis vaccine: a randomised, double-blind, phase 2b trial. Lancet. 2023;401(10379):843–55.
